# Supplementary material for: Prediction of prognosis in immunoglobulin a nephropathy patients with focal crescent by machine learning
Source: PLoS One. 2022 Mar 9;17(3):e0265017. doi: 10.1371/journal.pone.0265017 (PMC8906594; doi:10.1371/journal.pone.0265017)
Supplement: S1 Table — (DOCX) [file pone.0265017.s001.docx]

**S1 Table. Features included demographic, clinical, laboratory data and treatment of the IgAN patients.**

| Category | Feature name |
| --- | --- |
| Epidemiological characteristics | Age at biopsy, years |
|  | Gender |
| Clinical characteristics | Serum albumin , g/L |
|  | TP, g/L |
|  | Hematuria (red blood cells/high-power field) |
|  | Systolic blood pressure , mm Hg |
|  | Diastolic blood pressure , mm Hg |
|  | MAP, mm Hg |
|  | Serum creatine , mmol/L |
|  | Baseline eGFR , ml/min per 1.73m^2^ |
|  | Proteinuria , g/d |
|  | Serum cholesterol, mmol/L |
|  | Serum triglycerides, mmol/L |
|  | LDL-C, mmol/L |
|  | HDL-C, mmol/L |
|  | BUN, mmol/L |
|  | Serum IgA,g/L |
|  | Serum C3,g/L |
|  | Serum uric acid, mmol/L |
|  | Hypertension history, % |
|  | Hepatitis history, % |
|  | Disease course (months) |
| Pathological findings | Oxford M |
|  | Oxford E |
|  | Oxford S |
|  | Oxford T |
|  | Oxford C |
|  | Crescents proportion of glomeruli, % |
|  | Global crescent |
|  | Global crescent proportion of glomeruli,% |
|  | Cellular global crescent |
|  | Global crescent of fibrocyte |
|  | Global fibrous crescent |
|  | Segmental crescent |
|  | Cellular segmental crescent |
|  | Segmental crescent of fibrocyte |
|  | Segmental fibrous crescent |
| Treatments | Immunosuppression treatment |
|  | Renin-angiotensin system blockade treatment |

TP, total protein; MAP, mean arterial pressure; eGFR, estimated glomerular filtration rate; LDL-C, low density lipoprotein cholesterol; HDL-C, high density lipoprotein cholesterol; BUN, blood urea nitrogen; C3, [complement](D:/Program%20Files%20(x86)/Dict/8.9.9.0/resultui/html/index.html#/javascript:;) 3.
